# Differentially expressed long noncoding RNAs in RAW264.7 macrophages during *Brucella* infection and functional analysis on the bacterial intracellular replication

**DOI:** 10.1038/s41598-022-25932-6

**Published:** 2022-12-09

**Authors:** Xiang Guan, Hai Hu, Minxing Tian, Hongxu Zhuang, Chan Ding, Shengqing Yu

**Affiliations:** Shanghai Veterinary Research Institute, Chinese Academy of Agricultural Sciences (CAAS), 518 Ziyue Road, Minhang District, Shanghai, 200241 China

**Keywords:** Microbiology, Molecular biology

## Abstract

Long noncoding RNAs (lncRNAs) are a group of functional RNA molecules without protein-coding potential and play vital roles in majority of biological processes. To date, the expression profiles of lncRNAs and their influence on *Brucella* replication in RAW264.7 cells are poorly understood. In this study, we performed high-throughput transcriptome analysis to investigate the differentially expressed lncRNAs associated with *Brucella abortus* S2308 infection. Of these, 8, 6, 130 and 94 cellular lncRNAs were differentially expressed at 4, 8, 24 and 48 h post-infection, respectively. Moreover, 1918 protein-coding genes are predicted as potential *cis* target genes of differentially expressed lncRNAs by searching protein-coding genes located at upstream and downstream of lncRNA loci on the chromosome DNA of *Mus musculus*. Gene Ontology and Kyoto Encyclopedia of Genes and Genomes analyses indicated that majority of lncRNA target genes were associated with *B. abortus* infection. Fourteen lncRNAs from transcriptome data were selected for qRT-PCR verification, confirming 13 were differentially expressed. Animal experiments revealed three were differentially expressed in vivo by qRT-PCR analysis. Furthermore, knockdown of LNC_000428 by CRISPR/dCas9 inhibition or Locked Nucleic Acids transfection downregulated *Tnfrsf8* expression at mRNA level and increased *Brucella* intracellular replication. Thus, we provide a novel evidence that lncRNAs induced by *Brucella*-infection function on *Brucella* intracellular replication.

## Introduction

Long noncoding RNAs (lncRNAs) are a class of RNA molecules which are longer than 200 nucleotides and are poor at encoding proteins. High-throughput transcriptomic analysis revealed the primary transcripts and processed RNAs in several mammalian cells which are transcribed at 39% and 22% of the genome on average, respectively^1^. However, only a small proportion of primary transcripts are found to be processed into protein-coding RNAs and are responsible for 1.0–1.5% of genomes in mammalian cells, whereas most RNAs are classified lncRNAs and small noncoding RNAs^2,3^. Some recent reports have uncovered that human and mouse cells synthesize thousands of lncRNAs, and it is known that lncRNAs are more tissue-specific and less conserved than protein-coding genes^4,5^. Moreover, there is mounting evidence indicating that many lncRNAs serve as critical regulatory roles in the biological processes associated with the development, metabolism and immunization in mammals^6–8^. Additionally, host-cell-synthesized lncRNAs in response to pathogen infection are emerging as vital regulation factors to determine the fate of these pathogens, including both bacteria and viruses^9,10^. Therefore, profiling cellular lncRNAs on different infection situations could provide more detail in the cellular response to pathogen infection.

*Brucella*, which cause severe worldwide chronic zoonosis called brucellosis, are facultative intracellular Gram-negative pathogens^11,12^. *Brucella* are canonically classified into six species including *Brucella melitensis*, *B. abortus*, *B. suis, B. canis*, *B. ovis,* and *B. neotomae*^13^. Of the six species, *B. melitensis*, *B. abortus* and *B. suis* are more important causes of zoonosis, which seriously impact human health^14^. Evidence has shown that the pathogenesis of *Brucella* spp. is attributed to the bacterial surviving intracellularly within both phagocytic and non-phagocytic cells of its hosts^15,16^. To facilitate bacterial stealth in their host cells, several identified *Brucella* components play essential roles during infection, such as lipopolysaccharide, the type 4 secretion system and two-component regulatory systems^17^. In addition, host proteins in response to *Brucella* infection also show vital roles to regulate *Brucella* intracellular survival^18,19^. However, the profiles of macrophage-produced lncRNAs during *Brucella* infection are less described.

In this study, we performed high-throughput transcriptome analyses in S2308-infected RAW264.7 macrophages to discover the differentially expressed lncRNAs at 4, 8, 24 and 48 h post-infection (hpi). Potential *Cis* target genes of these lncRNAs were then predicted and subjected to GO and KEGG analyses for evaluation of their function following *Brucella* infection. Of these, three lncRNAs were further confirmed to be differentially expressed in *Brucella*-infected macrophages and mice by qRT-PCR analysis. Furthermore, LNC_000428 was found to be associated with *Brucella* intracellular replication in macrophages. Thus, this report provided an insight on exploring the functions of lncRNA on *Brucella* intracellular survival in their host cells.

## Results

### Transcript assembly and lncRNA identification

In this study, raw reads from RNA-Seq data were qualified to generate clean reads for assembly of transcripts. For each sample, the total numbers of clean reads were greater than 81,000,000 and base mismatch rates were 0.02%. Values of all Q20 and Q30 evaluating the probability of error on clean reads were also more than 90% (Supplementary Table 1). Moreover, the sizes of clean reads from RNA-Seq data in this study were more than 12 Gb (Supplementary Table 1) whereas the size of genome of *Mus musculus* is about 2.5 Gb^20^. All results support that the RNA-Seq was eligible for the following read alignments and transcript assembly. Subsequently, all clean reads were aligned to the genome of *Mus musculus* and assembled using the TopHat2 software^21^. The alignment results are displayed in Supplementary Table 2. More than 82% of total clean reads from RNA-Seq data from each group were completely aligned to the genome of *Mus musculus*. Additionally, at least 39% of total clean reads were arranged on the positive strands of chromosome DNA of *Mus musculus*, while greater than 38% reads were completely aligned on the negative strands (Supplementary Table 2). For the characterization of assembled transcripts in this study, a total of 3158 lncRNAs in RAW264.7 cells were screened (Supplementary Data 1).

### Characteristics of differentially expressed lncRNAs and their potential *cis* target genes

**Figure 1:**
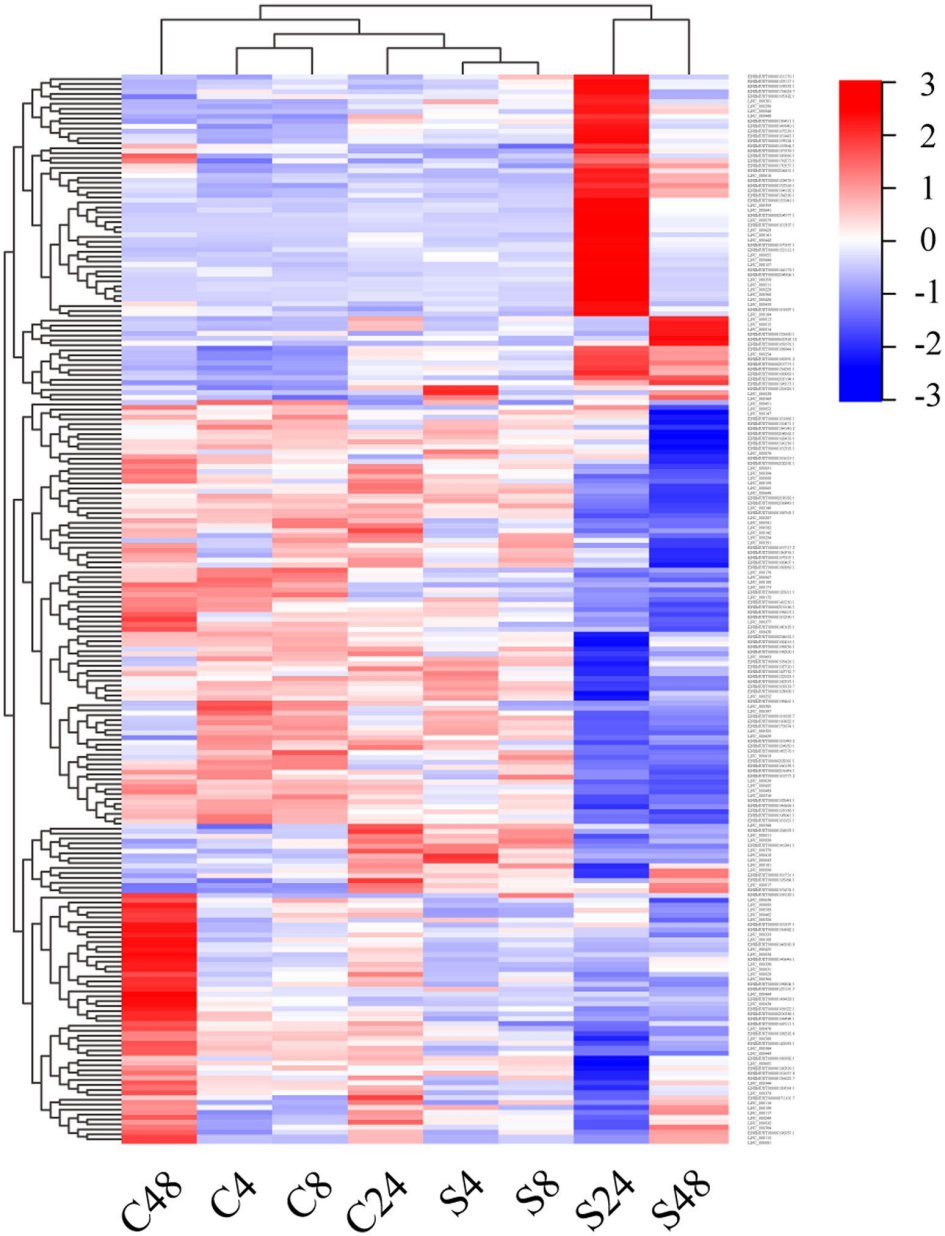
Hierarchical heat map of differential lncRNA expression induced by *Brucella* infection in RAW264.7 cells by RNA-Seq analysis. A hierarchical cluster heat map exhibiting RNA-Seq based differential expression of lncRNAs in RAW264.7 cells with a *Brucella abortus* S2308 infection for 4, 8, 24 and 48 h was generated by using the Pretty heatmaps (https://github.com/raivokolde/pheatmap). Both the red and blue colors represent higher and lower expression of lncRNA genes by log10 transformed expression values, respectively. S4, S8, S24 and S48 represent an S2308 infection for 4, 8, 24 and 48 h, respectively, while C4, C8, C24 and C48 represent mock infection for 4, 8, 24 and 48 h.

To identify the differentially expressed lncRNAs, expression of lncRNAs were quantified by fragments per kilobase of transcript per million reads sequenced (FPKM) by applying the Cuffdiff software^22^. Subsequently, determination of the differential expression of lncRNAs between infection and mock cells were performed with the Cuffdiff software. Results of the differential lncRNA expression were shown in Fig. 1. According to the analysis of RNA-Seq data in this study, 8, 6, 130 and 94 lncRNAs were differentially expressed in RAW264.7 cells with S2308 infection for 4, 8, 24 and 48 h, respectively (Supplementary Data 2). Afterwards, potential *cis* target genes of differentially expressed lncRNAs were predicted by searching protein-coding genes located at 100 Kb upstream and downstream of lncRNA loci on the chromosome DNA of *Mus musculus*, resulting in a total of 1918 coding genes as potential *cis* target genes for lncRNA (Supplementary Data 3).

### GO and KEGG annotation of the potential lncRNA target genes

**Figure 2:**
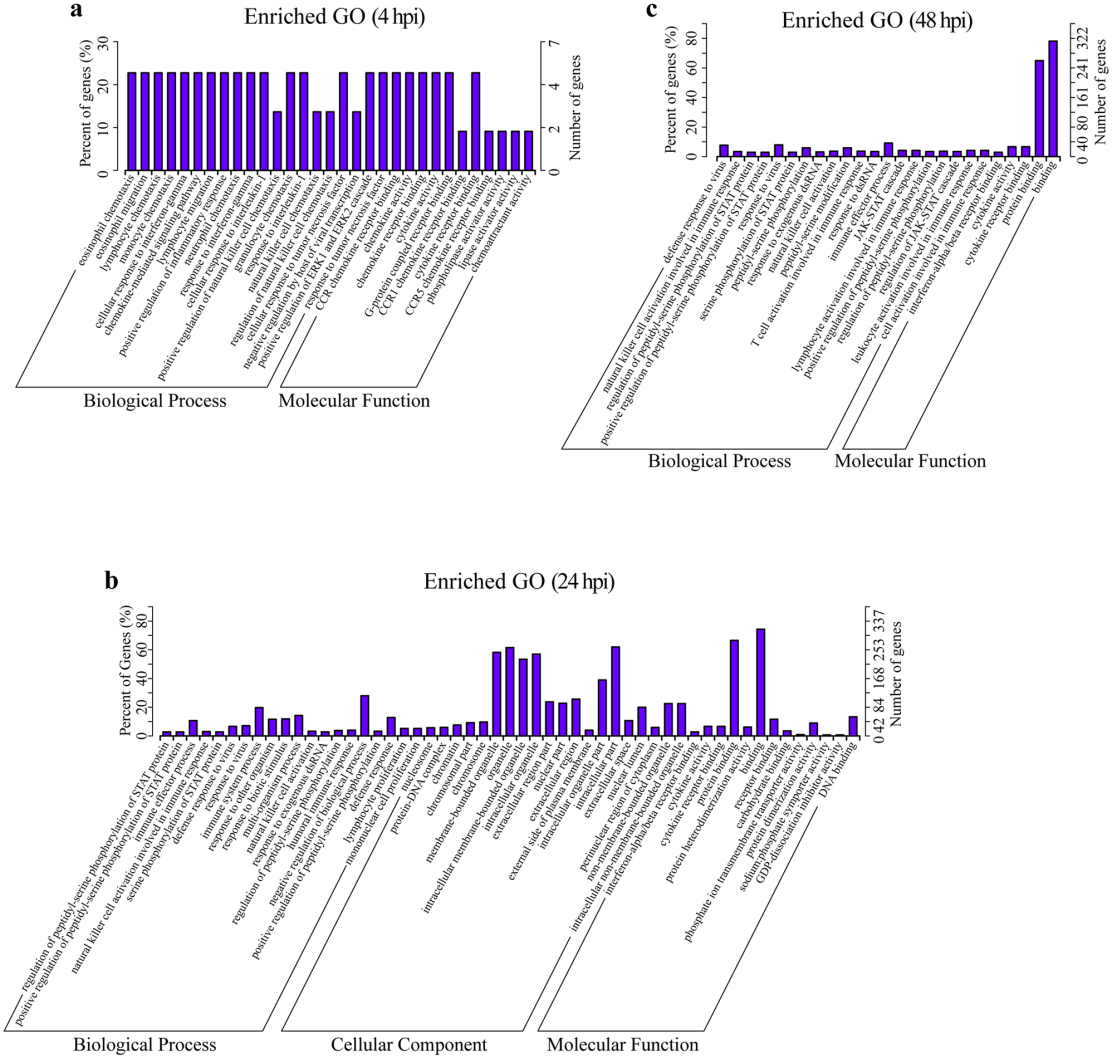
GO analysis of lncRNA target genes. GO analysis of *cis* predicted target genes of lncRNAs were differentially expressed in RAW264.7 cells with a *Brucella abortus* S2308 infection for 4 (**a**), 24 (**b**) and 48 (**c**) h, according to the corrected *p*-value (< 0.05). A total of 20 significant terms were shown and GO terms with no significance were enriched at 8 hpi. The *x-axis* indicates the GO terms under three subcategories: Biological Process, Cellular Component and Molecular Function. The left *y-axis* indicates gene percentages (numbers of enriched genes of each term divided by the total gene numbers), and the right *y-axis* indicates gene numbers for each term.

*Cis* predicted target genes of differentially expressed lncRNAs were investigated by GO analysis to reveal the ties between a gene product and its biological process, molecular function or cellular component. GO terms were classified into three subcategories including biological processes, cellular components and molecular function. According to the corrected *p*-value (*p* > 0.05) on GO enrichment, the top 20 GO terms with the most significance in three subcategories were shown in Fig. 2. From the results of the GO analysis of cis predicted target genes from differentially expressed lncRNAs induced by S2308 at 4 hpi, only GO terms in subcategories of biological process and molecular function were significantly enriched (Fig. 2a). The GO terms included a response to interferon-gamma (GO:0071346) and positive regulation of inflammatory response (GO:0050729) in biological processes, cytokine activity (GO:0005125) and cytokine receptor (GO:0005126) binding in molecular function. Significantly enriched GO terms at 8 hpi were not found in this study. At 24 hpi, negative regulation of biological (GO:0048519) and immune system processes (GO:0002376) were the two most dominant terms. For molecular function, binding (GO:0005488) and protein binding (GO:0005515) were most important; and the intracellular part (GO:0044424), organelle (GO:0043226), membrane-bounded organelle (GO:0043227), intracellular organelle (GO:0043229) and intracellular membrane-bounded organelle (GO:0043231) were highly represented in the cellular component (Fig. 2b). At 48 hpi, GO terms in each cellular component was not significantly enriched (Fig. 2c). The immune effector process (GO:0002252) and response to virus (GO:0009615) in biological processes, protein binding (GO:0005515) and binding (GO:0005488) of molecular function, were the two highly illustrated terms (Fig. 2c).

**Figure 3:**
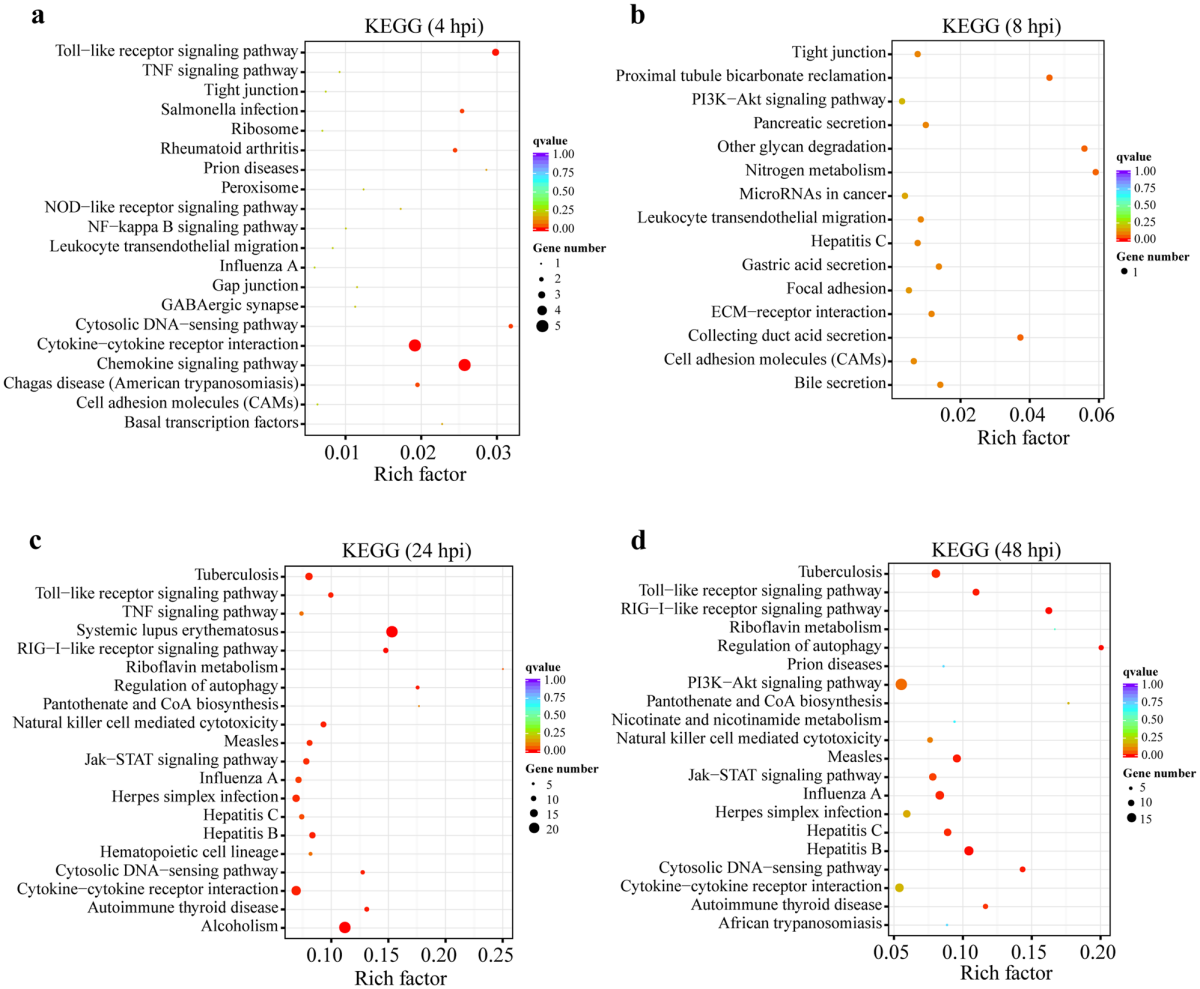
KEGG annotation of lncRNA target genes. KEGG pathway enrichment for *cis* predicted target genes of lncRNAs differentially expressed in RAW264.7 cells with infection of *Brucella* abortus S2308 for 4 (**a**), 8 (**b**), 24 (**c**) and 48 (**d**) h. A total of 20 significantly enriched pathways were shown in this figure and the Rich factor represents the ratio of lncRNA target genes to total annotated genes in a pathway (https://www.kegg.jp/kegg/kegg1.html).

KEGG enrichment on *cis* predicted target genes of all differentially expressed lncRNAs was also carried out. According to the corrected *p*-value, top 20 most significant pathways enriched in this report were shown in Fig. 3, including chemokine signaling pathway and cytokine-cytokine receptor interaction at 4 hpi (Fig. 3a), nitrogen metabolism and other glycan degradation at 8 hpi (Fig. 3b), systemic lupus erythematosus and alcoholism at 24 hpi (Fig. 3c), and RIG-I-like receptor signaling pathway and Hepatitis B at 48 hpi (Fig. 3d).

### Verification of differentially expressed lncRNAs by qRT-PCR

**Figure 4:**
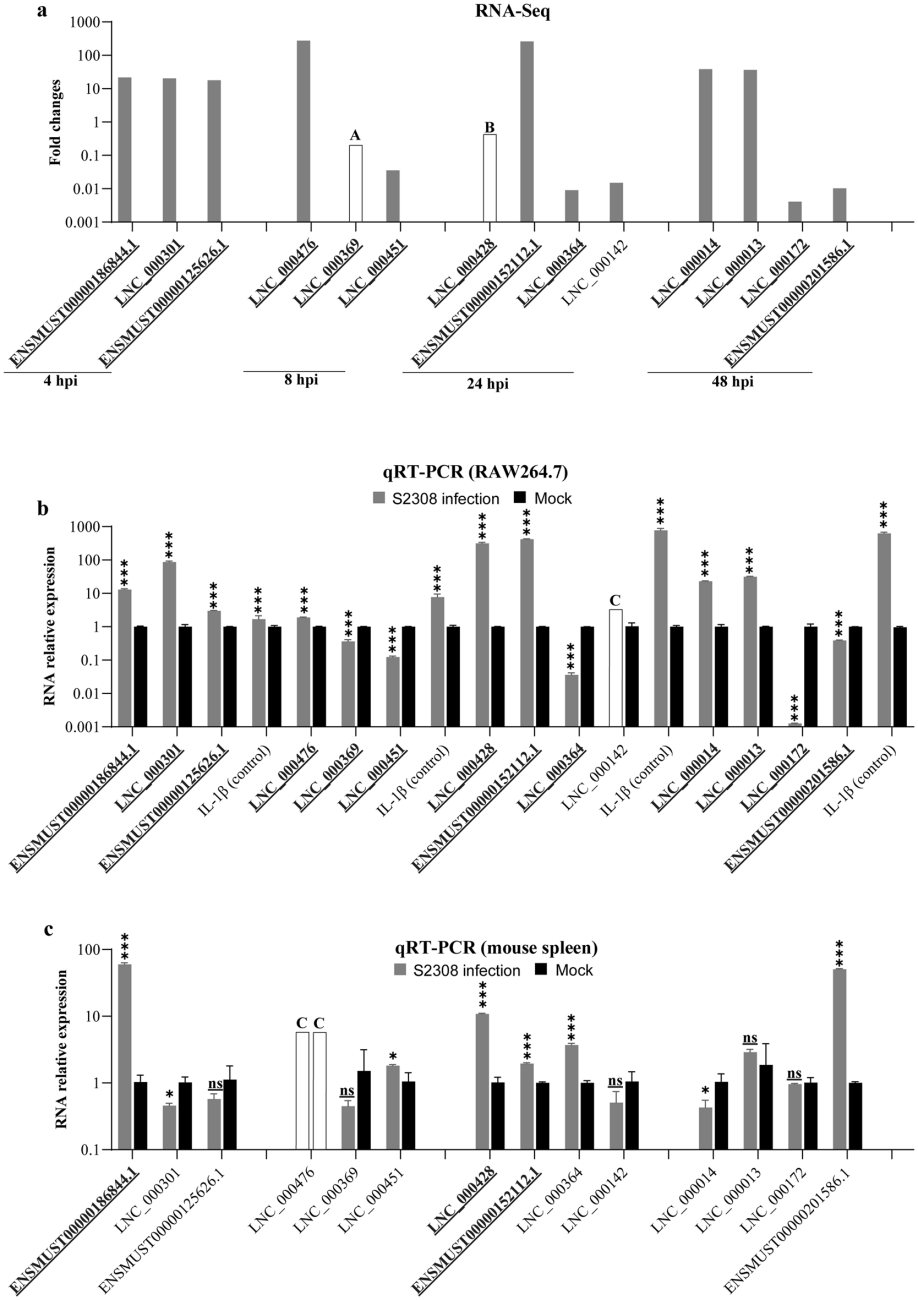
qRT-PCR validation of differentially expressed lncRNAs in S2308-infected RAW264.7 cells and mice. (**a**) 14 differentially expressed lncRNAs with greater than 16-fold changes found by transcriptomic analysis in RAW264.7 cells with infection of *Brucella abortus* S2308 for 4, 8, 24 and 48 h were selected for further verification. (**b**) The selected 14 lncRNAs were verified by qRT-PCR on RAW264.7 macrophages infected with S2308 at 1000 MOI for 4, 8, 24 and 48 h. Relative RNA expression was decided by normalizing the expression of lncRNA to *Gapdh*. Expression of IL-1β was used as the control for detection. (**c**) Expression of the 14 selected lncRNAs in the spleens of mice infected with S2308 were verified at 24 h post infection by qRT-PCR. Relative RNA expression was decided by normalizing the expression of lncRNA to *Gapdh*. Bold underlined gene names represent the differentially expressed lncRNAs, which were confirmed by RT-PCR. hpi: hours post-infection. A: Expression fold change in RNA-Seq data is infinitely great while the expression quantity in an infected group is approximately zero; B: The value in RNA-Seq data is infinitely great while the expression quantity in a mock group is approximate to zero; C: lncRNA was not applicable for detection by qRT-PCR. *P < 0.05; ***P < 0.001; ns, not significant.

From the results of RNA-Seq analysis, differential lncRNA expression between infection and control groups were verified by qRT-PCR. According to lncRNA expression with log2 transformed fold changes more than 4, the differential expression of a total of 14 lncRNAs from all timepoints was demonstrated by qRT-PCR through relative RNA expression in RAW264.7 cells infected with S2308 for 4, 8, 24 and 48 h (Fig. 4). Aside from LNC_000142, which was not detectable in this study (Fig. 4b), the qRT-PCR verified the differential expression of all 13 selected lncRNAs, which were similar to the data of the RNA-Seq analysis (Fig. 4a). Additionally, the differential expression of 14 selected lncRNAs was investigated by qRT-PCR in spleens of mice infected with S2308 at 24 hpi (Fig. 4c). The results showed that differential expression of the three lncRNAs, including ENSMUST00000186844.1, LNC_000428 and ENSMUST00000152112.1, was found in the spleens of mice in the infection and control groups, which was similar to the data from RNA-Seq analysis and qRT-PCR verification (Fig. 4).

### Knockdown of LNC_000428 promoted *Brucella* replication in macrophages and decreased *Tnfrsf8* expression at the mRNA level

**Figure 5:**
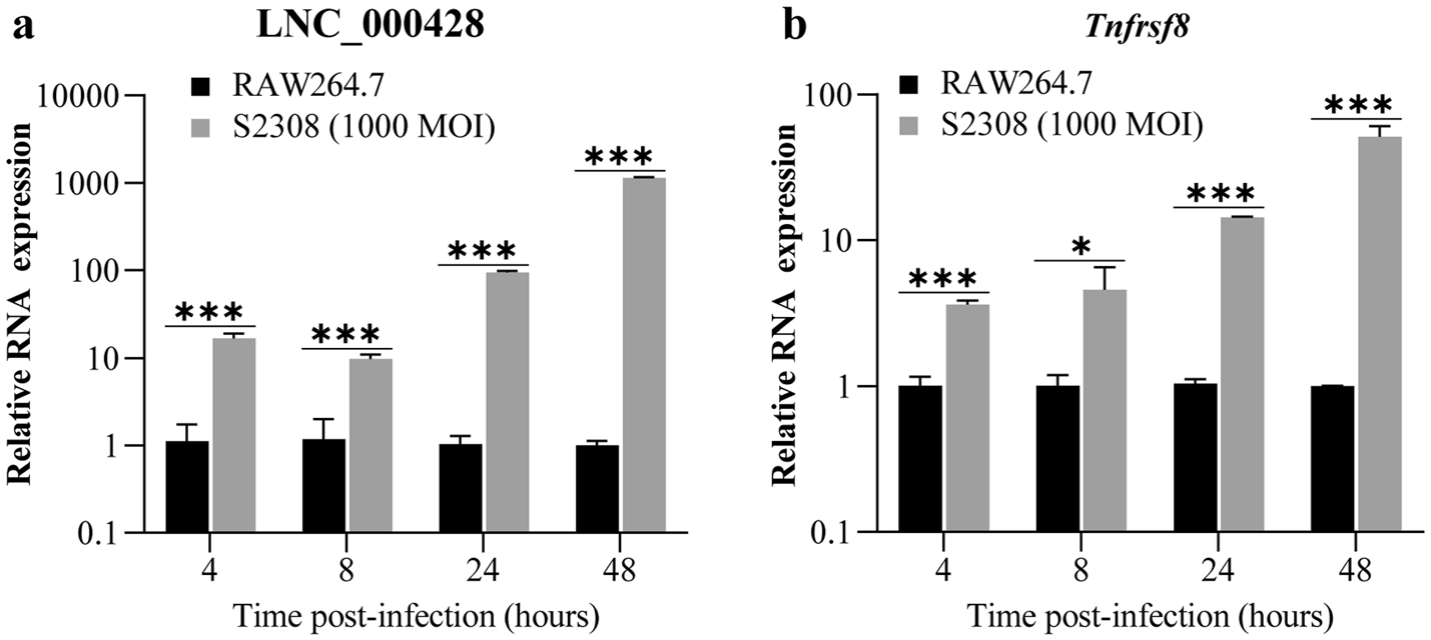
Spatio-temporal expression of LNC_000428 and its anti-sense gene *Tnfrsf8* in S2308-infected RAW264.7 cells determined by qRT-PCR. Expression of LNC_000428 (**a**) and *Tnfrsf8* (**b**) in RAW264.7 cells with a S2308 infection at a MOI of 1000 for 4, 8, 24 and 48 h were detected by qRT-PCR. Relative RNA expression was decided by normalizing the expression of lncRNA to *Gapdh*. *P < 0.05; ***P < 0.001.

Based on the prediction of the *cis* target gene for the differentially expressed lncRNAs in this report, LNC_000428 was an anti-sense lncRNA of *Tnfrsf8* on the chromosome 4 of *Mus musculus* (Supplementary Data 3). Therefore, spatio‐temporal expression of LNC_000428 and its anti-sense gene *Tnfrsf8* in RAW264.7 cells with S2308 infection, were determined by qRT-PCR. The results exhibited that differential expression of both LNC_000428 and *Tnfrsf8* were induced by S2308 infection in RAW264.7 cells, and that differential lncRNA expression at 48 hpi was definitely higher than the outcomes at 4, 8 and 12 hpi (Fig. 5). These results support that anti-sense lncRNA LNC_000428 could serve a critical role in *Brucella* infection to murine macrophages.

**Figure 6:**
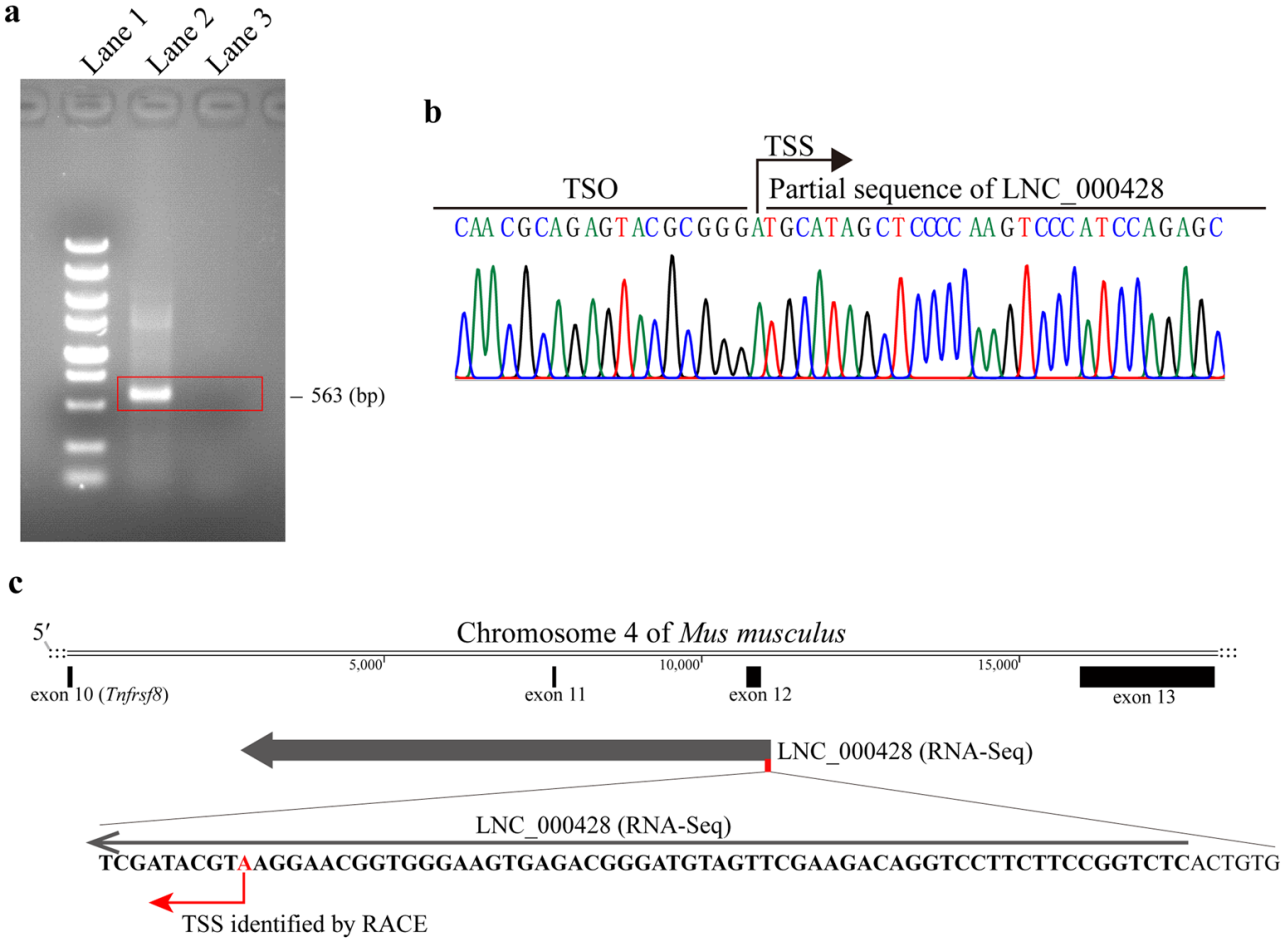
Determination of transcriptional start site (TSS) of LNC_000428 by 5′ RACE system. (**a**) The sizes of DNA products from RACE PCR determined by gel electrophoresis. Lane 1: DL5000 DNA marker (Vazyme); lane 2: RACE PCR of LNC_000428; lane 3: negative control of RACE PCR, its PCR template came from the reverse transcription reaction containing no reverse transcriptase. The original gel was displayed in the Supplemental Fig. 1. (**b**) The sequencing result of DNA products of RACE PCR. TSO = template-switching oligo. (**c**) Schematic draw of LNC_000428 TSS on the chromosome 4 of *Mus musculus* verified by 5′ RACE system.

**Figure 7:**
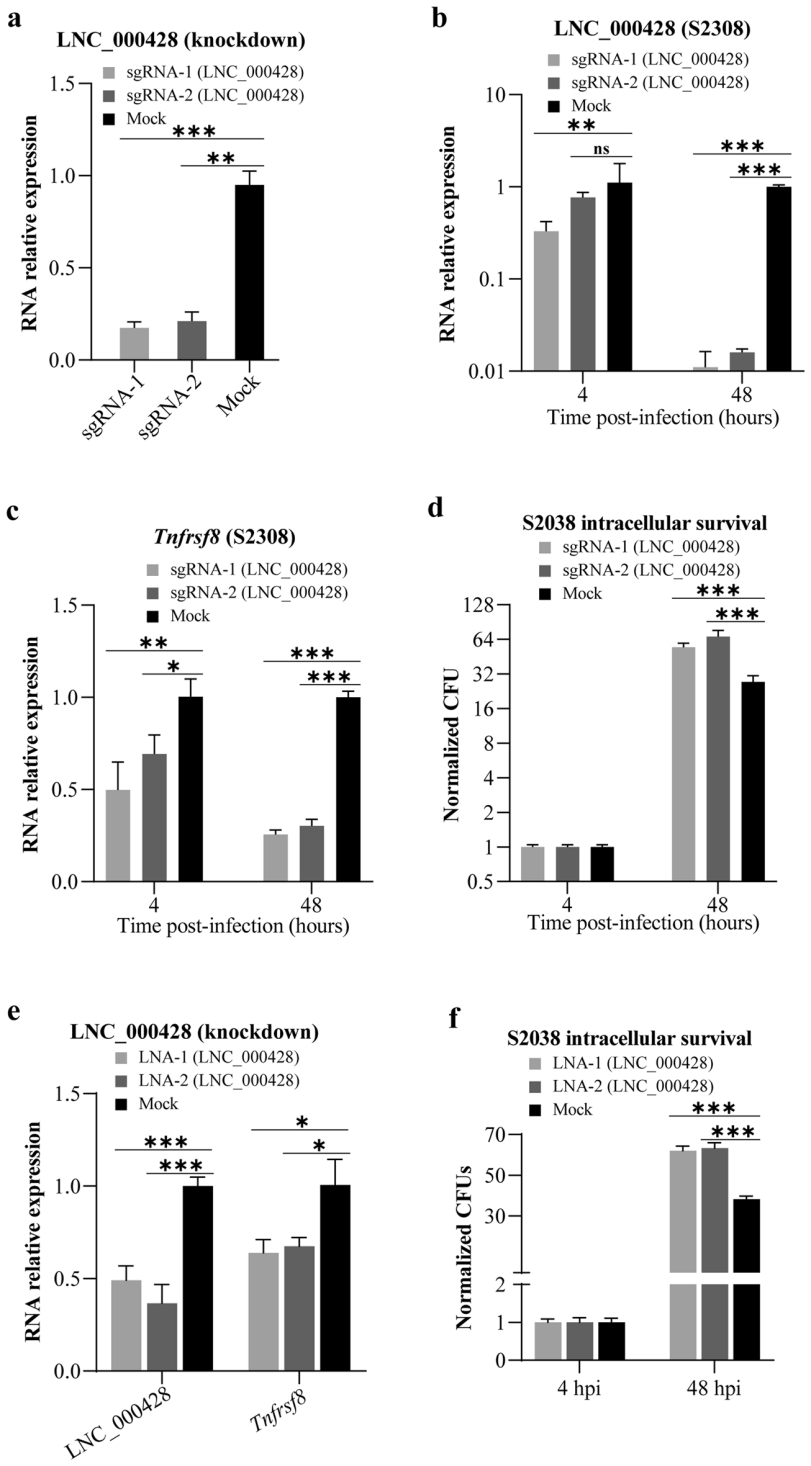
LNC_000428 knockdown in RAW264.7 cells impacting *Brucella*-induced *Tnfrsf8* expression and *Brucella* intracellular survival. (**a**) LNC_000428 expression in cell lines from which lncRNA expression in RAW2647 cells is knocked down by the CRISPR/dCas9 inhibition system. Expression of LNC_000428 (**b**) and *Tnfrsf8* (**c**) induced by S2308 infection in lncRNA knockdown cell lines was detected by qRT-PCR, while the cells were infected with S2308 at an MOI of 1000 for 4 and 48 h. Relative RNA expression was decided by normalizing the expression of lncRNA to *Gapdh*. (**d**) Impacts of LNC_000428 knockdown in RAW264.7 cells on *Brucella* intracellular survival were shown by determination of the intracellular bacterial with CFUs while cell lines were infected with S2308 at a MOI of 1000 for 4 and 48 h. All data at 48 hpi were normalized to 4 hpi when bacterial CFU at each time point was divided by mean of CFUs at 4 hpi. (**e**) Locked Nucleic Acids (LNAs) targeted to LNC_000428 were delivered into RAW264.7 cells by TurboFect Transfection Reagent. At 24 h post transfection, expression of LNC_000428 and *Tnfrsf8* were determined by qRT-PCR. (**f**) Impacts of LNC_000428 knocked down expression by LNAs in RAW264.7 cells on *Brucella* intracellular growth. Bacterial CFUs were monitored at 4 and 48 hpi. All data at 48 hpi were normalized to 4 hpi when bacterial CFU at each time point was divided by mean of CFUs at 4 hpi. *P < 0.05; **P < 0.01; ***P < 0.001. ns: not significant.

To investigate whether knocking down of LNC_000428 affects *Brucella* intracellular survival, a CRISPR/dCas9 inhibition (CRISPRi) system was employed in this report. Initially, a transcriptional start site (TSS) of LNC_000428 was identified by the 5′ RACE system (Fig. 6a, b), and was shown far away from the site of *Tnfrsf8* TSS. We confirmed that the LNC_000428 TSS was located at 59 bp downstream of the transcript from the results of RNA-Seq analysis (Fig. 6c). In addition, targeting sites of sgRNAs in CRISPRi system are highly closed the TSS of genes^23^. Subsequently, two sgRNAs targeting LNC_000428 were designed where sgRNA-1 was located at 51–70 bp upstream of the verified LNC_000428 TSS, and sgRNA-2 at 48–67 bp downstream. After the CRISPRi system was delivered into RAW264.7 cells by the lentivirus system, relative RNA expression by qRT-PCR revealed that LNC_000428 in the two cell lines containing the CRISPRi system were significantly reduced (Fig. 7a), supporting that the two sgRNAs were remarkably effective in the inhibition of LNC_00042 expression in RAW264.7 cells. Besides that, relative RNA expression of LNC_000428 and *Tnfrsf8* were significantly reduced in the two cell lines with an infection of S2308 for 48 h (Fig. 7b, c). Moreover, the knockdown of LNC_000428 in RAW264.7 cells increased the numbers of intracellular *Brucella* when the two lncRNA-reduced cell lines were infected with S2308 for 48 h (Fig. 7d). These results indicate that murine-derived anti-sense lncRNA LNC_000428 possesses a crucial role for *Brucella* intracellular survival in host macrophages.

LNA is a modified single stranded antisense nucleotide, which repress the gene expression via targeting lncRNA by sequence-specific Watson–Crick base pairing to induce cleavage by RNase H^24^. In this study, LNA was designed to be the antisense sequence of the lncRNA, because the lncRNA is also the antisense sequence of the coding gene, therefore, LNA will not complement and pair with coding genes, nor direct influence the function of the coding protein. Thus, gapmer LNAs targeting LNC_000428 were used to knock down LNC_000428 in RAW264.7 cells. LNC_000428 knockdown decreased the expression of *Tnfrsf8* (Fig. 7e). When LNC_000428 was knocked down by LNAs, intracellular *Brucella* S2308 in RAW264.7 macrophages were increased accordingly (Fig. 7f). Therefore, knocked down expression of LNC_000428 by CRISPRi or LNAs benefited *Brucella* intracellular growth in RAW264.7 macrophages, indicating that LNC_000428 could be a negative regulator for intracellular *Brucella* replication during infection.

## Discussion

*Brucella* spp. are facultative intracellular Gram-negative bacteria that can cause persistent infection by localizing in macrophages within animal bodies^25^. Brucellosis caused by *B. melitensis*, *B. abortus* and *B. suis* is a zoonosis that can be transmitted from animals to humans. *Brucella abortus* S2308, a smooth type recovered in 1940, is a highly virulent strain from an aborted fetus of a cow that had been in contact with cattle experimentally infected with a mixture of *B. abortus* cultures^26^. Since then, *B. abortus* S2308 has been diffusely applied as a reference, and a challenge strain has been applied to explore the pathogenic mechanisms in relation to brucellosis^27,28^. Reportedly, *Brucella* spp. can survive within resident macrophages such as RAW264.7 cells, a widely used macrophage cell line to investigate infection and immunity of *Brucella* in vitro^29,30^^.^ Studies are progressively showing that the entrance of smooth strains of *B. abortus* into macrophages and epithelioid cell lines is mediated by a lipid raft at the first step of infection, followed by generating a *Brucella*-containing vacuole (BCV)^31^. Following the nascent BCVs interacting with early endosomes, BCVs are converted into endosomal *Brucella*-containing vacuoles (eBCVs) by undergoing interaction with late endosomes and lysosomes at 0.5–2 hpi and up to 20 hpi^32^. Nearly 90% of internalized *Brucella* are killed in macrophages within the first 4 h^33^. At 8 hpi, eBCVs fuse with the endoplasmic reticulum to form replicative *Brucella*-containing vacuoles (rBCVs) where *Brucella* initiate replication^34^. Finally, autophagic Brucella-containing vacuoles (aBCVs) are formed at 48–72 hpi for bacterial release^35^. Although the landscapes of small noncoding RNAs and mRNAs in *Brucella*, as well as mRNAs, lncRNAs and microRNAs from RAW264.7 cells infected with *Brucella* were defined by RNA sequencing^36–39^, the profiling of the lncRNAs in macrophages from the early to late stages of *Brucella* infection was less known. In this study, we employed high-throughput sequencing and qRT-PCR verification to describe the differentially expressed lncRNAs in RAW264.7 cells infected with *B. abortus* S2308 from the early to late stages. The findings may strongly provide an insight to investigate whether lncRNAs served as regulators associated with intracellular survival of *Brucella* in macrophages.

In macrophages, it seems that evidence has revealed that *Brucella* could regulate Toll-like receptor signaling pathways, TNF signaling pathways and inflammation to facilitate bacterial survival in host cells^40,41^. During the infection of *M. tuberculosis*, lncRNAs were considered to regulate TNF expression in macrophages to impact the replication of bacteria^42,43^. Pathways associated with immune response were also significantly obtained from GO and KEGG analyses, such as Toll-like receptor signaling pathways, TNF signaling pathways and inflammation. Taken together, some of the differentially expressed lncRNAs induced by an S2308 infection may be associated with immune response in macrophages.

In vivo, *Brucella* exhibit a preference of localizing within macrophages for successful replication by a stealthy strategy, and high bacterial loads were found in the mouse spleens which were reportedly contained extensively abundant macrophages^44–49^. In this report, we demonstrated three differentially expressed lncRNAs from RNA-Seq data by qRT-PCR in *B. abortus* S2308 infected RAW264.7 cells and in mouse spleens. These validated lncRNAs might have potential as regulators during *Brucella* infection. Furthermore, *Tnfrsf8*, as a well-known CD30, was predicted as one of the targets of anti-sense lncRNA LNC_000428, which was upregulated in *B. abortus*-infected RAW264.7 cells at 24 hpi and mouse spleens at 24 h post-infection. CD30 was reportedly expressed in several cell types, including activated and memory Th cells, NK cells, macrophages and those cells located in the thymus medulla^50–52^. In vivo, the knockout of CD30 in mice decreased the immune response against *L. monocytogenes* and mycobacterial infection^50,52^. In addition, *M. avium* infection resulted in lower levels of CD4^+^ and CD8^+^ T cells, monocytes, and B cells in spleens of CD30 knockout mice that are susceptible to this pathogen. In this study, knocked down of LNC_000428 by CRISPRi or LNAs decreased expression of *Tnfrsf8* and increased *Brucella* intracellular growth in murine macrophages, suggesting its negative regulation on *Brucella* intracellular replication. Thus, LNC_000428 might be an immune-associated lncRNA and potentially play an important role in *B. abortus* infection, which required further investigation.

## Conclusions

Here, we employed a high throughput RNA-Seq to explore the differentially expressed lncRNAs from S2308-infected macrophages spanning *Brucella* intracellular life. Function analysis of the lncRNA targets showed that differentially expressed lncRNAs might be associated with *Brucella* infection and immunity. qRT-PCR results revealed that three lncRNAs were differentially expressed in *Brucella*-infected macrophages and mouse spleens. Moreover, LNC_000428 was found to be associated with intracellular replication of *Brucella*. Therefore, our results provide a new insight for further investigation of lncRNAs and its role on *Brucella* intracellular survival to uncover the stealthy strategy of this pathogen in macrophages.

## Material and methods

### Animal ethics statement

Mice were purchased from the Shanghai Laboratory Animal Center of Experimental Animals, Shanghai, China, and housed in cages at a controlled temperature of 28–30 °C under biosafety conditions, with water and food provided ad libitum. The animal health and behavior were monitored and recorded twice every day at 12 h interval during the study. The study was approved by the Institutional Animal Care and Use Committee of Shanghai Veterinary Research Institute and accomplished in strict accordance with the Guide for the Care and Use of Laboratory Animals instituted by Shanghai Veterinary Research Institute, the Chinese Academy of Agricultural Sciences (approval no: SHVRI-SD-2019–088). Mice were monitored for the clinical signs or death twice a day at a 12 h interval and sacrificed by carbon dioxide inhalation in the cage when sampling of the spleen or at the experimental endpoint. Criteria for determining the end point of animal experiment include: (1) animals do not drink or eat; (2) The body weight decreased by 20% of that before the experiment; (3) Dying or unable to move. The study is reported in accordance with ARRIVE guidelines (https://arriveguidelines.org).

### Cell lines and bacterial strain

Murine macrophage cell line RAW264.7 and human embryonic kidney 293 T cells were purchased from the American Type Culture Collection (ATCC) and maintained in Dulbecco's Modified Eagle Medium (DMEM, Thermo Fisher) with 10% fetal bovine serum (FBS, Biological Industries) in an incubator supplied with 5% CO_2_ at 37 °C.

*Brucella abortus* S2308 was purchased from the ATCC and cultured in *tryptic soy broth medium (TSB, Difco, NJ, USA)* at 37 °C, with a rotation at 200 rpm/min. All materials with live *B. abortus* were handled within a biosafety level 3 laboratory facility at the Chinese Academy of Agricultural Sciences, China.

### Macrophage infection

RAW264.7 macrophages were infected with *B. abortus* S2308 as described^53^. Briefly, RAW264.7 cells were incubated with DMEM-resuspended *Brucella* at a multiplicity of infection (MOI) of 1000 for 60 min at 37 °C after cells were twice washed with phosphate-buffered saline (PBS). Subsequently, RAW264.7 cells were twice washed with PBS and treated with DMEM containing 100 μg/mL gentamycin for 60 min at 37 °C to kill extracellular *Brucella*. Finally, RAW264.7 cells were sustained in DMEM medium containing 10 μg/mL gentamycin and 1% FBS. Samples were collected at 4, 8, 24 and 48 hpi for RNA isolation followed by RNA-Seq analysis and qRT-PCR verification.

### RNA isolation, library preparation and sequencing

Total RNA from RAW264.7 cells were isolated by TRIzol Reagent (Invitrogen, USA) according to the procedures. Afterwards, 3 μg of total RNA from each sample as input material was treated by Epicentre Ribo-zero rRNA Removal Kit (Epicentre, USA) to remove ribosomal RNA, and rRNA-depleted RNA was precipitated by ethanol. RNA fragmentation was carried out by applying divalent cations under elevated temperature in NEBNext First Strand Synthesis Reaction Buffer (5×) before the first strand cDNA was synthesized using a random hexamer primer and M-MuLV Reverse Transcriptase. Subsequently, the second strand of cDNA was synthesized by using DNA Polymerase I and RNase H whereas dTTP in dNTPs mixture was replaced by dUTP. DNA fragments without overhangs were removed by exonuclease and polymerase, and then adenylated at the 3′ ends and ligated by the NEBNext Adaptor with hairpin loop structure. The library fragments with preferentially 150–200 bp in length were purified with AMPure XPsystem (Beckman Coulter, Beverly, USA), followed by treatment with 3 μL USER Enzyme (NEB, USA) for 15 min at 37 °C and for 5 min at 95 °C before PCR was performed with Phusion High-Fidelity DNA polymerase, Universal PCR primers and Index (X) Primer. Finally, the quality of products purified by AMPure XP system was assessed on the Agilent Bioanalyzer 2100 system.

The libraries were sequenced on an Illumina Hiseq 2500 platform to generate 125 bp paired-end reads, and the index-coded samples were clustered on a cBot Cluster Generation System using TruSeq PE Cluster Kit v3-cBot-HS (Illumia, USA) according to the manufacturer’s instructions.

### Transcript assembly and lncRNA identification

Paired-end clean reads from raw data by removing reads with adapter, ploy-N and low quality reads, were aligned to the *Mus musculus* genome (https://www.ncbi.nlm.nih.gov/genome/?term=Mus+musculus) using TopHat v2.0.9^21^. The assembly of mapped reads was performed by employing both Scripture (beta2)^54^ and Cufflinks (v2.1.1)^22^ in a reference-based approach. Afterwards, lncRNAs were identified through five steps: (1) mono-exon transcripts with low abundance and reliability were removed; (2) transcript (> 200 bp) were screened from step 1; (3) transcripts overlapping with the genes from protein databases and lncRNA databases were separated by using Cuffcompare^22^; (4) based on Cuffquant^22^ analysis, new transcripts with abundance (FPKM < 0.5) from step 3 were deleted and known lncRNAs were filtered from above step; and (5) novel lncRNAs were identified through evaluating the coding potential of assembled transcripts by utilizing Coding-Non-Coding-Index (CNCI) (v2)^55^, Coding Potential Calculator (CPC) (0.9-r2)^56^, Pfam Scan (v1.3)^57^ and PhyloCSF (phylogenetic codon substitution frequency) (v20121028)^58^.

### Differential expression of lncRNAs and their putative target genes

The number of FPKM determined by Cuffdiff (V1.3.0) (http://cole-trapnell-lab.github.io/cufflinks/install/) was used to describe the expression abundance of lncRNA, followed by identifying differentially expressed lncRNAs (*P* < 0.05) caused by a *B. abortus* infection^22^. Target genes of lncRNAs with differential expression were predicted by searching coding genes between 100 kb upstream and downstream of lncRNAs (Cis).

### GO and KEGG

GO enrichment of target genes from differentially expressed lncRNA, were implemented by the GOseq R package (V2.12) (http://www.bioconductor.org/packages/2.12/bioc/html/goseq.html)^59^. GO terms are considered to be significantly enriched when a corrected *p*-value is less than 0.05. Simultaneously, the KOBAS (V.2.0) (http://kobas.cbi.pku.edu.cn/home.do) was applied to test the statistical enrichment of lncRNA target genes in KEGG pathways^60^.

### qRT-PCR

cDNA was synthesized by HiScript II Reverse Transcriptase (Vazyme) following the manufacturer's instructions, after total RNA was treated by TURBO DNA-free Kit (Invitrogen) to remove gDNA. Subsequently, qRT-PCR was performed by using ChamQ Universal SYBR qPCR Master Mix (Vazyme) according to the manufacturer’s protocol. The relative expression of different groups of genes was determined by quantifying the gene expression to the *Gapdh* mRNA level. The qRT-PCR primers were shown in Supplementary Table 3.

### Animal experiment

An animal model was used to perform experiments following the previous protocol^53^. Briefly, 10 healthy, non-immunized 6-week-old BALB/c female mice were divided into two groups (n = 2 groups). Five mice in one group (n = 5 mice) were placed in one cage, and 10 mice for two groups were placed separately in two cages (n = 2 cages). The random allocation of mice was performed by using the standard = RAND () function in Microsoft Excel. We tested 5 mice for each group is because we have to reduce the animal numbers as much as possible following the Guide for the Care and Use of Laboratory Animals instituted by Shanghai Veterinary Research Institute and ensure enough numbers for the subsequent effective statistical analysis. Each mouse in group 1 was intraperitoneally injected with 0.1 ml PBS containing no bacteria as the mock control, while each mouse in group 2 was injected intraperitoneally with 0.1 ml PBS containing 5 × 10^5^ CFU bacteria. At 24 h post-infection, each of the five mice from groups 1 and 2 were humanly sacrificed, and the spleens were collected and resuspended with TRIzol reagent followed by homogenization for RNA isolation according to the manufacturer's instruction. We kept the mice treatment order of PBS injection first (group 1), then bacteria injection (group 2) as the same with spleen collection order, mice in group 1 first followed by group 2, to minimize potential confounders. Five RNA samples from each group were prepared and combined for qRT-PCR analyses. Differentially expressed lncRNAs in spleens were measured using primers listed in Supplementary Table 3. The experiment was repeated three times.

### 5′ RACE system

The 5′ end of LNC_000428 was confirmed by applying the SMARTer RACE 5′/3′ Kit (TaKaRa), according to the manufacturer’s instructions. The primers involved in the RACE reaction were listed in Table 1 and other oligos were provided by the kit. Briefly, reverse transcription was carried out using a gene-specific primer, LNC_000428-RT. cDNA was used as template for RACE PCR with LNC_000428-GSP-1R and Universal Primer A Mix. Next, the PCR products cloned into the vector using 5 min TA/Blunt-Zero Cloning Kit (Vazyme, Nanjing, China) and the RACE PCR products, were identified by Sanger sequencing. Finally, the sequences of PCR products were aligned to genomic DNA of *Mus musculus* to obtain the transcriptional start site of LNC_000428.

**Table 1:** Oligos for RACE and knockdown of LNC_000428 in this study.

Iterms	Sequence (5′–3′)
**Oligos for RACE reaction**	
LNC_000428-RT	ATCAGATCAGAGTGCTAG
LNC_000428-GSP-1R	ATCAGCCACGGCTATATGGTGAG
**Oligos for lncRNA knockdown**	
Iterms	Sequence (5′–3′)
dCAS-F	AGTCGGTGCTTTTTTGAATTCGCTAGCTAGGTCTTGAAAGGAGTG
rdCAS-R	AGAGAAGTTTGTTGCGCCCTCGAGTACCAGCCAAGGTTCTTC
sgRNA-1F	**CACCG**GGCCAGAGTGACACTTCGTG
sgRNA-1R	AAACCACGAAGTGTCACTCTGGCC**C**
sgRNA-2F	**CACCG**TCTGCCCACATGATTAGACC
sgRNA-2R	AAACGGTCTAATCATGTGGGCAGA**C**
LNA-1	AGGAGGGTCAGAGGTTCA
LNA-2	AGTGGGTAGGTAGAGTGC

Bold and underlined: sticky ends for sgRNA cloning.

### CRISPRi- mediated lncRNA knockdown

To generate a CRISPRi vector, Cas9 on lentiCRISPR v2 (Addgene) was replaced by the DNA sequence containing dead Cas9 and Krüppel-associated box (a zinc-finger repressor protein). Briefly, fragment from lentiCRISPR v2 digested with *Xho*I and *Nhe*I was fused with the DNA sequence amplified from plasmid lenti-EF1a-dCas9-KRAB-Puro (Addgene), following the manual procedure of the ClonExpress Ultra One Step Cloning Kit (Vazyme). sgRNAs for CRISPRi-mediated lncRNA knockdown were designed from the website (https://chopchop.cbu.uib.no/), and cloned into the CRISPRi vector following the previous protocol^18^. The primers and oligos for sgRNA cloning were shown in Table 1. The CRISPRi system was delivered into target cells by lentivirus. For lentivirus packaging, three plasmids containing CRISPRi, psPAX2 (Addgene) and pMD2.G (Addgene) were co-transfected into 293 T cells using Lipofectamine 3000 Reagent (Thermo Fisher Scientific). After 6 h of transfection, the medium was replaced with DMEM containing 10% FBS. Supernatants containing lentivirus at 24 and 48 h after transfection were mixed and centrifuged at 600× *g* for 10 min at room temperature, followed by filtering with a 0.45 μ Millipore membrane. Finally, the lentivirus was aliquoted and stored at − 80 °C. To produce stable cell lines, RAW264.7 cells were infected with lentivirus. At 24 hpi, the medium was replaced with DMEM containing 10% FBS. At 48 hpi, RAW264.7 cells were treated with puromycin (5 μg/mL) for 6 days. Eventually, knockdown efficiency of CRISPRi was determined by qRT-PCR.

### Transfection of locked nucleic acids

In this study, Locked Nucleic Acids (LNAs) as antisense oligos of LNC_000428 were selected by using the software RNAstructure (V6.4) according to the recommended protocols. LNAs were delivered into RAW264.7 macrophages at a final concentration of 100 μM by TurboFect Transfection Reagent following the kit manual. At 24 h post transfection, the RAW264.7 cells were determined for the knocked down expression of LNC_000428 and *Tnfrsf8* by LNAs using qRT-PCR, then subjected to *Brucella* infection, and intracellular replication was determined at 24 hpi. LNAs sequences were provided in Supplementary Table 3.

### Intracellular survival of *Brucella*

RAW264.7 cells and lncRNA knockdown cell lines were infected with S2308 at 1000 MOI following the protocols described previously. At 4 and 48 hpi, cells were lysed with 0.5% Triton-100 in PBS, with incubation for 20 min at 37 °C. Next, lysates were serially diluted ten-fold and 100 μL dilutions were spread on TSA plates. The plates were incubated in an incubator supplied with 5% CO_2_ for 3 days at 37 °C. Ultimately, intracellular *Brucella* was designated with bacterial colony-forming units (CFUs) on the TSA plates. For normalization, total CFUs at 48 hpi were divided by total CFUs at 4 hpi.

### Statistical analysis

Analysis of differentially expressed lncRNAs by qRT-PCR as well as differential *Brucella* CFUs between groups was performed using a Student’s *t*-test (two-tailed) using Graph Pad Prism (version.8.3.0) (https://www.graphpad-prism.cn/). A *p*-value which is less than 0.05 was considered as being statistically significant. All data are presented as mean ± SD.

## Supplementary Information

Supplementary Information.

## Data Availability

The raw data that support the findings of this study are available from National Center for Biotechnology Information at https://www.ncbi.nlm.nih.gov/sra/PRJNA694452, reference number: PRJNA694452.
